# Chemotherapy-associated thromboembolic risk in cancer outpatients and effect of nadroparin thromboprophylaxis: results of a retrospective analysis of the PROTECHT study

**DOI:** 10.1186/1479-5876-9-179

**Published:** 2011-10-20

**Authors:** Sandro Barni, Roberto Labianca, Giancarlo Agnelli, Erminio Bonizzoni, Melina Verso, Mario Mandalà, Matteo Brighenti, Fausto Petrelli, Carlo Bianchini, Tania Perrone, Giampietro Gasparini

**Affiliations:** 1 Medical Oncology Department, Treviglio Hospital, P.le Ospedale N.1, Treviglio (BG), 24047, Italy; 2 Medical Oncology Unit, Oncology and Hematology Department, Ospedali Riuniti, Largo Barozzi N.1, 24128, Bergamo, Italy; 3 Internal and Vascular Medicine--Stroke Unit, University of Perugia, piazzale Menghini, Perugia, 06100 Italy; 4 Institute of Medical Statistics and Biometry, University of Milan, via Vanzetti N.5, Milan, 20100, Italy; 5 Medical Oncology Department, Cremona Hospital, Via Largo Priori N.1, Cremona, 26100, Italy; 6 Scientific Department, Italfarmaco S.p.A., Via dei Lavoratori, 54, Cinisello Balsamo (MI), 20092, Italy; 7 Oncology Department, San Filippo Neri Hospital, via Martinotti 20, Rome, 00135, Italy

**Keywords:** chemotherapy, nadroparin, prophylaxis, thrombosis, LMWH

## Abstract

**Background:**

Cancer patients receiving chemotherapy are at increased risk of thrombosis. Nadroparin has been demonstrated to reduce the incidence of venous and arterial thrombotic events (TEs) by about 50% in cancer outpatients receiving chemotherapy. The aims of this retrospective analysis were to evaluate the thromboembolic risk and the benefit of thromboprophylaxis according to type of chemotherapy.

**Methods:**

Cancer outpatients were randomly assigned to receive subcutaneous injections of nadroparin or placebo. The incidence of symptomatic TEs was assessed according to the type of chemotherapy. Results were reported as risk ratios with associated 95% CI and two-tailed probability values.

**Results:**

769 and 381 patients have been evaluated in the nadroparin and placebo group, respectively. In the absence of thromboprophylaxis, the highest rate of TEs was found in patients receiving gemcitabine- (8.1%) or cisplatin-based chemotherapy (7.0%). The combination of gemcitabine and cisplatin or carboplatin increased the risk to 10.2%. Thromboprophylaxis reduced TE risk by 68% in patients receiving gemcitabine; with a further decrease to 78% in those receiving a combination of gemcitabine and platinum.

**Conclusions:**

This retrospective analysis confirms that patients undergoing chemotherapy including gemcitabine, platinum analogues or their combination are at higher risk of TEs. Our results also suggest that outpatients receiving chemotherapy regimens including these agents might achieve an increased benefit from thromboprophylaxis with nadroparin. Clinical Trial registration number: NCT 00951574

## Background

Cancer patients are at increased risk of thrombosis [1]. Thromboembolic complications may be the first manifestation of malignancy and are associated with a high rate of morbidity and mortality [2]. Thromboembolic events (TEs) occur in 4-20% of patients with cancer [3]. Evidences of thrombosis have been reported in up to 50% of cancer patients in autoptic series [4]. TEs and infections are the second cause of death in cancer patients after the cancer itself [5].

Large studies have suggested that some solid malignancies including pancreatic, lung, colon, ovarian, primary hepatic and brain cancer are associated with a higher risk of TEs [6,7]. Risk factors, such as age, gender, bed-rest, venous catheters, surgery, radiotherapy and infections, also increase the risk of thrombosis in cancer patients [8].

Epidemiological studies have identified chemotherapy as an additional risk factor for a hypercoagulability state and thrombosis [9]. The pathophysiology of chemotherapy-related TEs may involve a variety of mechanisms that include enhanced procoagulant activity, reduced anticoagulant synthesis, stimulation of platelet aggregation and endothelial damage [10]. In a population-based study, chemotherapy was associated with risk of venous TEs that was increased 6.5-fold compared to non-cancer patients [11]. Platinum analogues, anthracyclines and fluoropyrimidines are agents mostly associated with a pro-thrombotic effect. In a prospective study, platinum-based regimens were significantly associated with venous TEs [12]. Even within this class of agents, rates of TEs seem to be higher in patients receiving cisplatin compared to oxaliplatin [13]. Gemcitabine has also been associated with remarkable thrombotic and vascular side effects [14]. Lastly, venous thromboembolic disease and catheter-related thrombosis have been documented in patients receiving 5-fluorouracil [15-18]. The use of concomitant steroids, erythropoietin preparations and granulocyte colony-stimulating factors (G-CSF) has also been associated with an increased incidence of TEs in cancer patients [9].

Recently, Agnelli et al [19] demonstrated that nadroparin almost halved, from 3.9% to 2.0%, the absolute rate of thromboembolic complications. The overall amount of reduction in symptomatic outcomes is consistent with those attributable to low-molecular-weight heparin in prevention of venous thromboembolism in other clinical settings [20].

The PROTECHT (PROphylaxis of ThromboEmbolism during CHemoTherapy) study highlighted chemotherapy as an independent risk factor for thromboembolism in a wide cancer population and defined the benefit of thromboprophylaxis [21]. The PROTECHT results have not the impact to change current practice for TE prophylaxis in the overall ambulatory cancer population receiving chemotherapy, otherwise a selection of patients The aims of this retrospective analysis were to identify subgroups of patients at high risk of TEs, stratified according to the type of chemotherapy, who could have an enhanced benefit from TEs prophylaxis.

## Patients and methods

### Design of the PROTECHT Study

The PROTECHT (PROphylaxis of ThromboEmbolism during CHemoTherapy) was a randomized, group sequential, placebo-controlled, double-blind, multicentre, clinical outcome study (NCT 00951574) [19]. Outpatients with metastatic or locally advanced solid tumours were randomized in a 2:1 ratio to receive either subcutaneous injections of nadroparin (3800 anti-Xa IU once daily) or placebo. Study treatment was started on the same day as chemotherapy and was given for the duration of chemotherapy or up to a maximum of 120 days (+/- 10 days). The primary outcome was the composite endpoint of symptomatic venous or arterial thromboembolic events, as adjudicated by an independent committee. Major bleeding was the main safety outcome.

Retrospective analysis of the PROTECHT study population, according to type of chemotherapy and concomitant medications, was carried out. All cytotoxic agents used in ≥ 1% of patients were evaluated. All concomitant medications that might potentially interact with coagulation factors were evaluated: steroids, G-CSF, nonsteroidal anti-inflammatory drugs, aspirin, erythropoietin, blood and related products [9].

An evaluation of the TE risk according to Khorana score [22] has been evaluated. This predictive score assigns 2 points to very high risk cancer sites (pancreatic or gastric) or 1 point to high risk cancer sites (lung, ovarian or bladder). In addition, 1 point is assigned for each of the followings: platelet count > 350 × 109/L, hemoglobin < 10 g/dl and/or use of erythropoietin-stimulating agents, leukocyte count > 11 × 109/L and body mass index > 35 kg/m2. Patients with a score ≥ 3 are at high risk to develop TEs

The study was done in accordance with the provisions of the Declaration of Helsinki and local regulations. The protocol was approved by the institutional review board at each study centre, and written inform consent was obtained from all patients before randomization.

### Statistical Methods

Descriptive statistics refer to all included patients. For continuous variables, the mean, standard deviation, median, minimum and maximum values were calculated. For each discrete variable, the number of cases in each category, in relation to all cases with non-missing values of that variable, was calculated.

Venous and arterial TE rates, grouped by the type of chemotherapy regimen, were reported as risk ratios (RRs) with associated two-tailed 95% CI. The approach adopted for the sub-group analyses consisted in considering findings as hypothesis-generating as a guide to prioritize additional studies. Hence, no adjustment of type I error rate was adopted for the multiplicity of confidence intervals over sub-groups. All statistical calculations were carried out using SAS version 9.1.

## Results

Overall, 1150 patients were included in the primary efficacy and safety analyses of the PROTECHT study. Fifteen of the 769 patients treated with nadroparin (2.0%) and 15 of the 381 patients treated with placebo (3.9%) had a thromboembolic event (P = 0.024). Five patients in the nadroparin (0.7%) and none in the placebo group had major bleeding (P = 0.18). The two study arms were well balanced for demographic characteristics and cancer site. Patient characteristics are listed in Table 1. Most patients were female (51.7%, 595/1150) and chemotherapy naïve (53.7%, 618/1150), with a median age of 64 years (range 27-84). Among the 1150 evaluable patients, the most common cancers were gastrointestinal 36.5% (420/1150), lung 24.3% (279/1150), breast 14.3% (165/1150) and ovarian 12.4% (143/1150). The median number of chemotherapy cycles was 4 in both placebo and treatment groups.

**Table 1:** Patient characteristics.*

	Nadroparin (*N = 769*)	Placebo (*N = 381*)
**Age **(years)		
Mean ± SD	62.1 ± 10.3	63.7 ± 9.2
Median (Min - Max)	64.0 (27-83)	65.0 (36-84)
**Sex**		
Female	51.6 (397)	52.0 (198)
Male	48.4 (372)	48.0 (183)
**Cancer Site**		
Gastrointestinal^a^	35.4 (272)	38.8 (148)
Lung	25.9 (199)	21.0 (80)
Breast	14.3 (110)	14.4 (55)
Ovary	12.5 (96)	12.3 (47)
Pancreas	4.7 (36)	4.5 (17)
Head and Neck	2.5 (19)	4.5 (17)
Other	4.8 (37)	4.5 (17)
**Disease Stage**		
Advanced	69.8 (537)	72.2 (275)
Metastatic	30.2 (233)	27.8 (106)
**Chemotherapy naïve**		
Yes	52.7 (405)	55.9 (213)
No	47.3 (364)	44.1 (168)
**Number of chemotherapy cycles**		
Mean ± SD	3.2 ± 1.4	3.4 ± 1.5
Median (Min - Max)	4.0 (1-7)	4.0 (1-13)
**Central venous catheter**		
Yes	41.9 (322)	38.6 (147)

* Data are % (N) unless otherwise indicated. ^a^Gastrointestinal (colon, rectum, stomach)

According to Khorana risk score patients at high risk of TEs are equally distributed among the two arms: 8.6% and 11.1% in the nadroparin and placebo group, respectively (Table 2).

**Table 2:** Khorana risk score and Khorana risk group evaluated among the PROTECHT patients

	Nadroparin (N = 765*)	Placebo (N = 378*)
**Khorana Risk score**		
0	38.2 (292/765)	36.8 (139/378)
1	32.2 (246/765)	30.4 (115/378)
2	21 (161/765)	21.7 (82/378)
3	8 (61/765)	9.8 (37/378)
4	0.5 (4/765)	1.1 (4/378)
5	0.1 (1/765)	0.3 (1/378)
Khorana Risk group*		
High risk	8.6 (66/765)	11.1 (42/378)
Intermediate risk	53.2 (407/765)	52.1 (197/378)
Low risk	38.2 (292/765)	36.8 (139/378)

Data are % (N) unless otherwise indicated. *For 4 patients in the nadroparin group and 3 patients in the placebo group evaluation of Khorana risk score has not be perform due to missing information for the variables(site of cancer, platelet count, haemoglobin level, leukocyte count and body mass index).

In the nadroparin arm, chemotherapy regimens containing vinca alkaloids were statistically more frequent (11.7%, 90/769) than in the placebo arm (7.1%, 27/381), but no other differences were found for the other chemotherapeutic regimens.

Concomitant medications that could potentially interact with blood coagulation were equally balanced in both groups. The most common concomitant medication was steroids (77%; 886/1150), granulocyte colony-stimulating factor (G-CSF) (15.2%; 175/1150), nonsteroidal anti-inflammatory drugs (NSAID) (11.7%; 135/1150) and erythropoietin (7.8%; 90/1150). Data regarding chemotherapy and concomitant medications by treatment group are shown in Table 3.

**Table 3:** Chemotherapy and concomitant medications by treatment group

	Nadroparin (n = 769) % (n)	Placebo (n = 381) % (n)	P-value (two tailed)
**Chemotherapy regimen containing^a^:**			
5-Fluorouracil	37.1 (285)	39.6 (151)	0.40
Cisplatin	23.0 (177)	22.6 (86)	0.87
Gemcitabine	20.3 (156)	22.6 (86)	0.37
Oxaliplatin	18.6 (143)	23.4 (89)	0.06
Docetaxel	18.5 (142)	17.6 (67)	0.72
Carboplatin	15.5 (119)	14.4 (55)	0.64
Irinotecan	12.5 (96)	10.8 (41)	0.40
Vinca alkaloids	11.7 (90)	7.1 (27)	0.01
Capecitabine	7.9 (61)	7.9 (30)	0.97
Epirubicin	7.0 (54)	6.3 (24)	0.65
Etoposide	5.3 (41)	4.5 (17)	0.53
Cyclophosphamide	4.3 (33)	4.7 (18)	0.74
Liposomal doxorubicin	3.8 (29)	2.9 (11)	0.44
Adriamycin	3.3 (25)	5.0 (19)	0.15
Trastuzumab	3.0 (23)	2.4 (9)	0.54
Cetuximab	0.9 (7)	0.8 (3)	0.83
Bevacizumab	0.5 (4)	0.5 (2)	0.99
**Concomitant Medication ^b^**			
Steroids	76.2 (586)	78.7 (300)	0.34
G-CSF	15.1 (116)	15.5 (59)	0.86
NSAID	11.2 (86)	12.9 (49)	0.41
Erythropoietin	7.4 (57)	8.7 (33)	0.46
Blood and related products	2.7 (21)	3.9 (15)	0.27
Aspirin	2.2 (17)	2.1 (8)	0.90

^a^The most used antineoplastic drugs (≥ 1%) are reported in the table; the sum is not 100% of study population; ^b^Concomitant medications that could potentially interact with coagulation factors; the sum is not 100% of study population

Abbreviation: G-CSF, Granulocyte colony-stimulating factor; NSAID, Nonsteroidal anti-inflammatory drugs

### Retrospective analysis results

In the absence of thromboprophylaxis (placebo group), the highest rate of TEs was found in patients receiving gemcitabine (8.1%; 7/86) or cisplatin (7.0%; 6/86). In the small subset receiving etoposide or epirubicin, the rate of TEs was 11.8% (2/17) and 8.3% (2/24), respectively. In patients treated with 5-fluorouracil containing regimens, TEs occurred in 3.3% of cases (5/151) (Table 4).

**Table 4:** Proportion of symptomatic TEs according to the type of chemotherapy regimen

	% (n/N)		(95% CI)
**Thromboembolic events**	**Nadroparin**	**Placebo**	**Relative risk**

Overall PROTECHT population	2 (15/769)	3.9 (15/381)	0.5 (0.24-1.00)
**Chemotherapy regimen containing:**			
5-Fluorouracil	2.5 (7/285)	3.3 (5/151)	0.74 (0.24-2.30)
Cisplatin	2.3 (4/177)	7.0 (6/86)	0.32 (0.09-1.12)
Gemcitabine	2.6 (4/156)	8.1 (7/86)	0.32 (0.09-1.05)
Oxaliplatin	0.7 (1/143)	1.1 (1/89)	0.62 (0.04-9.83)
Docetaxel	1.4 (2/142)	4.5 (3/67)	0.31 (0.05-1.84)
Carboplatin	0.8 (1/119)	5.5 (3/55)	0.15 (0.02-1.45)
Epirubicin	0.0 (0/54)	8.3 (2/24)	ND
Adriamycin	0.0 (0/25)	5.3 (1/19)	ND
Irinotecan	3.1 (3/96)	0.0 (0/41)	ND
Vinca alkaloids	2.2 (2/90)	3.7 (1/27)	0.60 (0.06-6.36)
Capecitabine	0.0 (0/61)	3.3 (1/30)	ND
Etoposide	2.4 (1/41)	11.8 (2/17)	0.21 (0.02-2.14)
Cyclophosphamide	0.0 (0/33)	5.6 (1/18)	ND
**Previous Chemotherapy**			
Naïve	2.5 (10/405)	5.2 (11/213)	0.48 (0.21-1.11)
Non-naïve	1.4 (5/364)	2.4 (4/168)	0.58 (0.16-2.12)

No TE events were observed among patients receiving trastuzumab, cetuximab, bevacizumab, liposomal doxorubicin or mitomycin. Abbreviation: ND, not determinable.

Among platinum agents (cisplatin, carboplatin and oxaliplatin), the highest rate of TEs was found in cisplatin containing regimens (7.0%; 6/86), followed by carboplatin (5.5%; 3/55) and oxaliplatin (1.1%; 1/89) (Table 4). The addition of gemcitabine to platinum compounds (cisplatin or carboplatin) increased the rate of TEs to 10.2% (5/49) (Table 5). When vinca alkaloids were combined with platinum compounds, the risk increased to 9.1% (1/11) (Table 5).

**Table 5:** Proportion of symptomatic TEs in patients receiving a platinum-based regimens (cisplatin or carboplatin) in combination with gemcitabine, docetaxel or vinca alkaloids

	% (events/pts)		(95% CI)
Platinum-based regimen*	**Nadroparin**	**Placebo**	**Relative risk**

Without gemcitabine	1.5 (3/201)	4.5 (4/89)	0.33 (0.08-1.45)
With gemcitabine	2.2 (2/90)	10.2 (5/49)	0.22 (0.04-1.08)
Without docetaxel	2.2 (5/224)	6.5 (7/108)	0.34 (0.11-1.06)
With docetaxel	0.0 (0/67)	6.7 (2/30)	N.D.
Without vinca alkaloids	1.6 (4/252)	6.3 (8/127)	0.25 (0.08-0.82)
With vinca alkaloids	2.6 (1/39)	9.1 (1/11)	0.28 (0.02-4.15)

*Platinum-based regimen: cisplatin or carboplatin

Antithrombotic prophylaxis with nadroparin reduced the risk of developing a TE in comparison to placebo in all chemotherapy regimens (Table 4). In particular, thromboprophylaxis reduced the risk of TEs in gemcitabine- (RR, 95%CI: 0.32, 0.09 - 1.04), carboplatin- (RR, 95%CI: 0.15, 0.02 - 1.45) and cisplatin- (RR, 95%CI: 0.32, 0.09 - 1.12) -containing regimens (Table 4). A relatively lower reduction in the risk of TEs was seen in 5-fluorouracil- (RR, 95%CI: 0.74, 0.24 - 2.3), vinca alkaloids- (RR, 95%CI: 0.60, 0.06 - 6.36) and oxaliplatin- (RR, 95%CI: 0.62, 0.04 - 9.83) -containing regimens.

Thromboprophylaxis also reduced the risk of TEs (RR, 95%CI: 0.22, 0.04 - 1.08) in patients treated with the combination of gemcitabine and platinum compounds, and in patients treated with vinca alkaloids and platinum compounds (RR, 95%CI: 0.28, 0.02 - 4.15) (Table 5).

The rate of TEs in patients treated with concomitant medications known to potentially interact with blood coagulation has been evaluated (Table 6). In patients administered steroids, thromboprophylaxis reduced the risk of TEs by 40% (RR, 95%CI: 0.60, 0.06 - 6.36).

**Table 6:** Proportion of symptomatic TEs according to concomitant medication

	% (n/N)		(95% CI)
Concomitant Medication	Nadroparin	Placebo	Relative risk
Steroids	2.4 (14/586)	4.0 (12/300)	0.60 (0.28-1.28)
G-CSF	0.0 (0/116)	1.7 (1/59)	ND
NSAID	1.2 (1/86)	0.0 (0/49)	ND
Erythropoietin	3.5 (2/57)	3.0 (1/33)	1.16 (0.11-12.29)
Blood and related products	0.0 (0/21)	6.7 (1/15)	ND
Aspirin	5.9 (1/17)	25.0 (2/8)	0.24 (0.02-2.23)

Abbreviation: ND, not determinable; G-CSF, granulocyte colony-stimulating factors; NSAID, nonsteroidal anti-inflammatory drugs

## Discussion

Chemotherapy is well known to be an independent risk factor for development of TEs in cancer patients [23-26].

In the PROTECHT study [19], nadroparin was shown to reduce the absolute rate of clinically overt TEs by about 50% in cancer outpatients receiving chemotherapy for metastatic or locally advanced solid tumors in comparison to placebo. Despite the results of the PROTECHT study thromboembolic prophylaxis could not be reasonable for the whole ambulatory cancer patients receiving chemotherapy. To improve the risk-benefit ratio of thromboprophylaxis, clinicians should identify patients at higher risk of TEs, who could have more benefit from anticoagulant administration. The aim of our retrospective analysis has been to identify in the PROTECHT population which subgroups of patients were at higher risk of TEs, stratified according to the type of chemotherapy, who could have an enhanced benefit from TEs prophylaxis.

Our results suggest that cancer outpatients receiving chemotherapy in the absence of thromboprophylaxis (placebo group) had a high incidence of TEs during treatment with gemcitabine, cisplatin or carboplatin. Etoposide and epirubicin showed also a high rate of TEs, but in a small subgroup of patients. Among platinum agents, cisplatin and carboplatin showed a higher risk of TE complications in comparison to oxaliplatin. Combination therapy of gemcitabine with platinum-compounds (cisplatin or carboplatin) further increased the risk of TEs. Additionally, the combination docetaxel with platinum compounds appeared to increase the risk of TEs, although the sample size was too small to make any definitive conclusions.

The thromboembolic risk of cisplatin and gemcitabine has been previously described [14,15]. In vitro studies have demonstrated that cisplatin activates platelets, mononuclear cells and endothelial cells, which together may result in a prothrombotic state [27]; however, the exact role of gemcitabine in the activation of the coagulation cascade and haemostasis remains unknown [14]. Published case series, isolated reports and observational studies have suggested that gemcitabine, particularly if combined with cisplatin, increases the risk of TEs [28-30]. Cisplatin is already known as a chemotherapy drug with a higher thromboembolic risk in comparison to oxaliplatin [13,31]. Recently, Moore and colleagues published a large retrospective analysis which confirmed an unacceptable incidence of TEs in cancer patients receiving cisplatin-based chemotherapy [32].

At the time of the enrolment in the PROTECHT study, targeted therapies were not commonly used in cancer patients. In fact, only 4.2% (48/1150) of patients were treated with trastuzumab, cetuximab or bevacizumab. Nonetheless, it is worthwhile noting that the CALGB 80303 trial [33] recently compared bevacizumab plus gemcitabine versus gemcitabine alone in patients with advanced pancreatic cancer. In that trial, the rates of grade 3/4 venous thrombosis was similar in both arms (14% and 15%, respectively) suggesting that the role of gemcitabine in cancer-induced thrombosis could be prevalent. Of note that currently, the efficacy of thromboprophylaxis in patients receiving antiangiogenic agents remains an open questions to be evaluated in well-designed randomized trials.

According to published data [8], the rate of TEs is more than two-fold higher in chemotherapy naïve patients (5.2%, 11/213) compared to non-naïve patients (2.4%, 4/168). Nadroparin prophylaxis shown a trend in reduction for thromboembolic risk by 52% and 42% in naïve and non-naïve patients, respectively.

The results in the global PROTECHT population have shown that thromboprophylaxis reduced the risk of developing TEs by 48.8% compared to placebo ([19]. Considering the subgroup of patients receiving cisplatin or carboplatin, thromboprophylaxis reduced the risk of thrombotic complications by 68% and 85%, respectively. Thromboprophylaxis reduced risk of thromboembolic complications in patients receiving gemcitabine by 68%, further decreasing to 78% when gemcitabine was combined with platinum-compounds.

Our retrospective analysis has an exploratory purpose. Subgroup samples have not the adequate statistical power to detect clinically meaningful differences as statistically significant and to adjust p-values for the multiplicity across subgroups [34]. For the same reason, a multivariable analysis testing the interaction between subgroups and study treatment cannot be performed because such a statistical model would be clearly over-parameterized. Nevertheless and despite the aforementioned limitations [35] these data could be useful for stratifying the TE risk in ambulatory cancer population, when planning controlled clinical studies.

In conclusion, our results suggest that patients receiving gemcitabine, cisplatin or carboplatin or their combination are at increased risk of TEs. The clinical benefit of thromboprophylaxis with nadroparin, in outpatients receiving chemotherapy, could be even more evident when gemcitabine was combined with platinum-compounds containing regimens.

## Competing interests

CB and TP are employees of Italfarmaco S.p.A., Italy. GA is consultant of advisory board for Bayer, Boheringer, Sanofy Aventis. All other authors have no financial or other conflict to declare. This study was supported by Italfarmaco S.p.A. Milan Italy

## Authors' contributions

SB and GG contributed equally to this work. EB planned and reviewed the statistical analysis. All authors read and approved the final manuscript.
